# Barriers to Substance Use Treatment for Individuals with Substance Use Disorders

**DOI:** 10.21203/rs.3.rs-3693809/v1

**Published:** 2023-12-15

**Authors:** Joseph Robert Hudson, Jennifer Manuel

**Affiliations:** New York University; University of Connecticut

**Keywords:** Substance Use, Public Health, Stigma, Residential Treatment, Barriers, Help-Seeking, Treatment Access

## Abstract

**Background:**

Although residential substance use treatment has been shown to improve substance use and other outcomes, most with substance use disorders (SUDs) never seek professional treatment. Much research has been done on the barriers to seeking treatment. However, greater understanding is needed of the similarities and differences in the perceptual barriers to treatment held by clients and staff.

**Methods:**

This paper (1) identifies and compares adult client vs. staff perceptions of barriers to substance use treatment, and (2) compares perceptions between an urban vs. rural treatment setting. Secondary analysis of transcripts of semi-structured interviews with clients (n = 61) and staff (n = 37) from a residential substance use treatment program in New York (urban) and in Virginia (rural). Transcriptions of interviews were formally analyzed by two analysts using framework analysis.

**Results:**

The major results indicate that personal barriers (83%) were cited more frequently than interpersonal (15%) and structural barriers (24%). Staff were more likely to cite interpersonal barriers (19% vs. 11%) and structural barriers (29% vs. 20%) than were clients.

**Conclusions:**

These findings further demonstrate that personal culpability and self-blame are often felt by those with SUDs and this sentiment is often reinforced by treatment providers. Interventions are needed that can reduce the stigma of SUD’s, resulting in a shift away from the perception that barriers to treatment exist primarily at the personal level.

**Trial registration:**

The Office of Research Subjects Protection at Virginia Commonwealth University (approval #HM15020) and the University Committee on Activities Involving Human Subjects at New York University (approval #FY2016–56) approved the study procedures for the Virginia and New York studies, respectively.

## 1. Background

A chronic-care approach is increasingly being used in the treatment of substance use disorders (SUDs) - comparable to that of diabetes, hypertension, and asthma [1]. Nevertheless, rates of treatment access for SUDs remain low. According to the Results from the 2021 National Survey on Drug Use and Health [2], 43.7 million people over the age of 12 needed treatment in the U.S. Of that number, only three million (6.9%) received treatment at a specialty facility during the previous 12 months.

Previous qualitative research has identified a variety of frameworks within which to categorize the barriers to persons with SUDs obtaining professional treatment. These studies have contributed to a better understanding of service use experiences from the perspectives of different sample populations – women who self-managed change [3], adolescents [4], African- American women [5], adults with co-occurring substance use disorder and mental illness (COD) [6], rural clients and stakeholders [7], and alcohol and drug users in other countries [8, 9, 10]. Fewer studies have compared and contrasted barriers from multiple perspectives across diverse populations and settings. One exception to this limited research is a study examining barriers among three different groups: youth in substance use treatment, their parents, and treatment providers [4]. Findings suggest that while staff reported that denial among youth and parents was a common barrier to adolescents seeking treatment, parents cited previous failed attempts, involvement in the legal system, or challenges finding appropriate treatment as barriers to adolescents finding treatment due [4]. All youth, parents, and staff reported barriers to starting treatment, primarily due to lack of motivation and waiting lists for services [4].

This study builds upon existing research to identify and compare adult client vs. staff perceptions of barriers to substance use treatment and to compare perceptions between an urban vs. rural treatment setting. Data generated through this research will lead to a greater understanding of the perceived barriers within and between these populations, which can enhance the strength of the therapeutic relationship between clients and treatment professionals and contribute to decision-making processes that will improve access to professional treatment.

## 2. Methods

### 2.1. Design

This is a secondary analysis of two qualitative studies that identified perceived barriers, facilitators and treatment strategies of residential treatment providers and individuals with substance use disorders from two residential substance use treatment programs. One treatment program received public and private funding in central Virginia, and the second was publicly funded in New York City. The Office of Research Subjects Protection at Virginia Commonwealth University (approval #HM15020) and the University Committee on Activities Involving Human Subjects at New York University (approval #FY2016–56) approved the study procedures for the Virginia and New York studies, respectively.

### 2.2. Participants

Individuals engaged in treatment at two residential substance use treatment programs, along with the staff members from these programs who provided treatment to these individuals, were invited to participate in a 45- to 90-minute semi-structured interview. Twenty-nine individuals and 20 providers at the central Virginia site and 32 individuals and 17 providers at the New York City site were interviewed. All individuals were eligible if they were 18 years of age or older and enrolled in residential substance abuse treatment. Due to limited resources, non-English speaking individuals were excluded from participating in the study. Individuals were excluded if they were judged by the residential treatment team to be in immediate crisis (e.g., suicidal) or those with an acute or chronic mental disorder that interferes with their ability to provide consent. All staff worked in residential substance use treatment and represented direct-care treatment providers, supervisors, and directors.

### 2.3. Recruitment and data collection procedures

Our recruitment strategy for both the Virginia and New York individual samples included posting flyers in the residential program. Individuals who expressed interest in the study met with research staff who explained the purpose and voluntary nature of the study. Research staff provided the Virginia sample of individuals with a consent form, which was read aloud, and written consent was obtained prior to enrollment. For the New York sample, research staff provided individuals with an information sheet, which was read aloud, and obtained verbal consent from participants. A waiver of written consent was requested and approved for the New York sample, given that the only record linking the participant and the research would have been the consent document. Recruitment and data collection procedures lasted approximately three months, from October 2013 to December 2013, for the Virginia study, and approximately four months, from May 2015 to August 2015, for the New York study.

Trained research interviewers conducted in-person individual interviews at residential facilities for all individuals. All interviewers had at least a master’s degree or higher and prior research experience in qualitative methods. The interviewers received training in recruitment, semi-structured interviewing, data management, and ethics and safety. The principal investigator held weekly meetings with interviewers to assess progress and troubleshoot challenges experienced during the interviews.

Interviewers used a semi-structured interview guide that included questions on participants’ substance use treatment experiences and help-seeking behaviors; their service needs; and barriers and facilitators to their recovery post-discharge from residential substance use treatment. Related to the topic of help-seeking, staff and individual participants were asked, ‘What do you think keeps people from seeking/getting substance abuse treatment when they first need it?’ and were asked additional questions if further elaboration was warranted. The interviews lasted between 45 and 90 minutes in length. Individuals received a $30 gift card for their participation in the interview.

### 2.4. Data analysis

Interviews were audio-recorded and transcribed verbatim. We analyzed interviews with Dedoose software, which aids in the coding and organizing of qualitative data [11]. Using framework analysis, we used the socio-ecological model to categorize and explore personal, interpersonal, and structural factors that impede and facilitate using substance use treatment services. The socio-ecological framework posits that an individual’s health and behavior both shape and are shaped by factors at multiple levels: individual factors (i.e., race/ethnicity, age, housing); interpersonal factors (i.e., family and friendships); organizational factors (program-related issues); and structural factors (i.e., funding, regulations) [12]. The socio-ecological model is used because it highlights the complexities of barriers to treatment-seeking at multiple levels and considers how multiple layers of influence intersect to shape a person’s health and behavior.

The first author served as the primary analyst coding every transcript, while the second author, a senior researcher with expertise in qualitative analysis, served as a secondary analyst who reviewed the coding decisions of the primary coder. Consensus was sought to ensure consistent and reliable data and to discuss and resolve disagreements in coding. After coding was completed, the primary and secondary analysts reviewed the coded text in its entirety and refined codes through an iterative process in which we organized data into broad categories and then modified the categories as the analysis continued. We grouped the themes into three logical levels: personal, interpersonal, and structural levels. Once we finalized the themes, the first author ensured that only one answer per person per theme was counted to ensure that no theme was overrepresented. Descriptive statistics were conducted to calculate the frequencies and percentages of each theme endorsed by staff and individual participants. Participants often cited more than one theme; therefore, the total percentage of responses is greater than 100% of the number of participants. Descriptive analyses were conducted on each barrier to identify significance of differences between clients and staff. We used Fisher’s exact test, an appropriate test in the analyses of contingency Tables 2× 2 and larger with small sample sizes [13].

## 3. Results

The sample characteristics of clients and staff are summarized in Tables 1 and 2, respectively. A thematic analysis of data is summarized in Table 3. Participants often cited more than one of the subcategory barriers that fell under each of the main category of barriers; therefore, the total percentage of subcategory responses is greater than 100%.

### 3.1 Personal barriers to seeking treatment.

Participants expressed doubt as a barrier, through responses that indicate an overall lack of belief and trust that treatment would be effective.

One urban client stated: *‘You could look at statistics and see how many people really succeed and come out of treatment and stay clean and live a productive life versus how many people just go right back. I think that is discouraging.’*

Staff members agreed. For example, one staff member stated: *‘I guess you, you have to have the understanding that it’s not a lot of people that actually come out of these residential programs and make it...’*

Failure to recognize and acknowledge that one’s substance use warrants treatment was at the heart of the responses that pointed to denial as a barrier to treatment.

Clients responded: *‘Not realizing what was the truth kept me...When you start maturing and getting older and you realize that you can’t sit around and smoke weed and do drugs all day and you can’t live a productive lifestyle...’*

Staff added: *‘The disease itself. It’s a disease that tells you, you don’t have a disease. It tells you, you don’t have a problem.’*

Self-choice was mentioned more frequently (49%) by all participants than any other barrier at the personal level. This was expressed in responses that indicate the belief that those who fail to seek treatment are actually choosing to not seek treatment based on personal level of readiness.

Some clients attributed this choice to a feeling of self-reliance. One client reported: *‘Well some people feel they don’t need it. So sometimes you feel like you can do it by yourself. Like in my case I felt like I could do it without treatment.’* Other clients believed the choice was rooted in wanting to maintain the status quo, adding: *‘Even though they know they need the help they don’t want to disrupt the family lifestyle so they think they can hold it off or hold it together.’* Several clients pointed to the need to hit ‘rock bottom’ before they would make the choice to seek treatment – as if they had to experience a sufficient amount of negative consequences in order to be motivated to seek treatment.

While staff members concurred with the concept of self-choice as a barrier, they tended to most frequently point to the need for clients to experience a ‘rock bottom’ before choosing treatment. One staff member stated: *‘If they don’t hit their bottom they don’t seek treatment...if the cons don’t outweigh the pros then they won’t seek treatment.’*

Twenty-eight percent of all participants held a perception that an attachment to alcohol/drugs – needing the substance to cope/survive – was another key barrier to seeking treatment.

Some clients pointed out the strong hold that the substance had on them, saying: *‘The only thing you do turn to is the drug because it turns into your companion. It doesn’t tell you nothing, it soothes your pain, and it keeps you there and you can be there whenever you need it. So, the drug is filling a void. It’s the missing link that you think you’re missing.’*

Staff members also pointed to the self-medicating attachment to the substance, saying: *‘People that use substance abusers [are] like runners, they like, run away, so how they run away from their feeling, they medicate their selves.’*

Participants described how the negative societal perception of SUDs leads to internalized guilt, shame, and embarrassment to seek treatment – a barrier we defined as stigma.

Consistent with the views of others, one client stated*: ‘Shame, guilt...I can’t see anything else that would stop them from...they embarrassed. They have low self-esteem.’*

Staff members concurred with the idea of embarrassment, evidenced by responses such as: *‘The next barrier is that, it’s a big stigma. So, you know, to admit that you have a problem and to go into a treatment is that mean that you’re weak.’*

Participants also held the belief that those with SUDs are often unaware of treatment options/process. Although, within personal barriers, this was the least-often cited, still 11% of participants pointed to this lack of knowledge of the available treatment options and what to expect from the treatment process as being a barrier to seeking treatment.

One client expressed: *‘They don’t want to, or probably they don’t know that there is help out there. They don’t have enough resources.’*

Staff members agreed, with one staff member reporting: *‘I think that first of all many don’t know what resources are available or how to get referred or some of them don’t know that they can self-refer.’*

### 3.2 Interpersonal barriers to seeking treatment

Statements were classified as interpersonal barriers when participants spoke of negative social support. Like the views held by others, one client highlighted the peer pressure people often face, stating: *‘Pride, peer pressure, because their friends are all getting high. Now you figure that you want to stop, but they’re like, ‘Why you gone stop? We can all be high. Come on back, buy two’...’*

Staff members’ responses acknowledged the difficulty for individuals who live in environments wherein substance use is a normal part of life. One staff member stated: *‘I think the population I have worked with, it has been primarily that this is the normal behavior for the society in which they generally spend time, is to use.’*

### 3.3 Structural barriers to seeking treatment

While personal barriers and interpersonal barriers are more subjective in nature, structural barriers – cited by 24% of participants – are more concrete.

Some participant responses spoke to a general inaccessibility of treatment. One client pointed to the physical inaccessibility of treatment, stating: *‘I was sick, and it was easier to use than to go to the hospital. The same drugs they were giving me in the hospital, I was getting on the street.’ Another client brought attention to the lack of financial access to treatment, reporting: ‘Money and resources were the main issue. I had health insurance and that helped me into treatment at first but um, uh, you know. Like after care and all that stuff, I started reaching a cap on my insurance and then I paid for it.’*

Staff members agreed, but also pointed out the ways a lack of finances can create an indirect barrier to treatment, saying: *‘A lot of times it’s not having an ID or verifiable address. In order to get those you have to have a birth certificate, which they sometimes don’t have, mostly don’t have. In order to get those you need cash.’*

Ten percent of rural participants cited admission difficulty compared to only 2% of urban staff and clients. Clients reported being eager to enter treatment, but unable to do so because of waitlists, represented in the following statement by one client: *‘Probably waitlists. That was the biggest thing. ... I’m sure you’ve probably heard that from more than one person. A lot of people.’*

Staff members were aware of waitlists being a barrier, but also brought attention to the idea that admission prerequisites can also present a barrier to treatment, adding: *‘On our assessment sheets we require that they have their PPD’s and their blood pressure. ... they can’t be on any benzos or anything because you can’t take suboxone with benzos. But some of them are on methadone and they have to be below 30mg or less. So we run into a problem because I have to have them tapered down to that amount before I can bring them in here.’*

Nine percent of participants indicated that, based on one’s family and work responsibilities, treatment may seem unfeasible. Female clients were more aware of family responsibilities as a barrier, evidenced by one client stating: *‘A lot of women don’t come to treatment...because they got kids and who are they going to leave their kids with, and how are they going to see their kids if you’re not going to support them?’* Male clients often pointed to job insecurity as a barrier, stating: *‘I didn’t want to miss work, for real. Work was part of it. And I didn’t know if they were going to hold my job. That was the biggest thing...’*

Staff members also focused on family responsibilities, stating: *‘But – so some people have elderly family or elderly relative or, you know, someone they have to care of, or they’ve always been a, a caregiver – and that may stop them from coming in because they need to be there for their family.’* Another staff member highlighted the need to address the barrier of childcare, sharing: *‘The other thing we are providing when they are here receiving services is childcare...you can bring children up through 12 so that won’t be a barrier to getting the help that you need.’*

## 4. Discussion

Clients and staff from both urban and rural locations were consistent in citing barriers at the personal level more frequently than the interpersonal and structural levels. These findings suggest that, unlike with other chronic diseases, personal culpability and self-blame are often felt by those with SUDs, and this sentiment may be reinforced by treatment providers. However, clients were quicker to point to self-critical themes like denial, self-choice, and attachment to alcohol/drugs, whereas staff members chose less pathologizing personal barriers, such as stigma, doubt, and being unaware of treatment options. This phenomenon suggests the presence of internalized stigma in client participants, resulting in a greater amount of guilt and shame for not seeking treatment. ‘Self-choice’ was mentioned more frequently by all participants than any other barrier, which may be indicative of an internalized perception that individuals with a SUD *would* engage in treatment if they simply were ‘ready’ to do so. Another possible explanation lies in the fact that, prior to treatment, more urban clients were homeless, suggesting that other needs, such as housing, lead clients into treatment.

Staff pointed to the interpersonal barrier of negative social support more often than clients. It is important to not identify a SUD client as the sole target unit of treatment but to delve into the way that clients operate on inter- and intrapersonal levels and how they navigate their social relations. Considering social relationships in accessing SUD treatment is important because research has demonstrated that individuals with a SUD history with access to positive social support are less likely to relapse [14] compared to clients without such relationships.

Among structural barriers, ‘inaccessibility of treatment’ was mentioned most often by all staff and clients, indicating a unified perception among all participants that a lack of physical and/or financial access to treatment is the most common structural barrier. Rural clients were uninsured more often than urban clients and more urban clients were publicly insured compared to rural clients. These findings suggest that rural individuals may face greater challenges in both physically and financially accessing professional treatment. Staff cited structural barriers more often than clients, which is consistent with our earlier suggestion that clients perceive more self-critical barriers to treatment, while staff members have a greater awareness of other societal factors involved.

Several limitations warrant consideration when interpreting these findings. First, our sample in each of the geographic regions came from one residential treatment setting and thus may not be representative of all residential programs in urban and rural communities. Due to the qualitative nature, our findings are not designed to produce generalizable findings and caution should be used given the limited sampling. Future research with larger samples from multiple treatment sites may provide additional insights into the factors that these participants perceive as barriers to help-seeking in SUD treatment. Additionally, there is a risk that the data analysts’ beliefs and values may have influenced the results. However, we believe this risk is minimal given that analysts were not involved in direct service provision or supervision of services in the residential treatment settings, and because data analysis involved an iterative process for defining and refining themes. Finally, this study used data collected between 2013 and 2015 and may not be representative of the current patient population. However, at present, few comparative studies of urban and rural clients and providers exist.

## 5. Conclusion

Despite these limitations, the current study provides insight into the role of personal, interpersonal, and structural barriers to SUD treatment from the diverse perspectives of urban and rural clients and staff, with a greater emphasis placed on structural barriers among staff and rural participants and self-critical themes among clients. Future longitudinal research is needed to improve our understanding of barriers accessing SUD treatment across different settings and populations. Interventions are needed that can reduce internalized and public stigma of SUDs – the effects of which will result in a shift away from the perception that barriers to treatment exist primarily at the personal level and allow for greater focus, at the policy level.

## Figures and Tables

**Table 1:** Characteristics of individuals receiving residential substance use treatment services (n = 61)

	Richmond Clients (n = 29)	NY Clients (n = 32)

	*n (%) or M± SD*	*n (%) or M± SD*

Gender	13 (45.9)	9 (28.1)
Female	16 (55.2)	23 (71.9)
Male		

Age (years)	35.1 ± 09.6	41.1 ± 9.5

Race/Ethnicity	2 (06.9)	3 (09.4)
Other	6 (20.7)	14 (43.8)
Black/African American	7 (24.1)	11 (34.4)
Hispanic	14 (48.3)	4 (12.5)
White/Caucasian		

Education	14 (48.3)	13 (40.6)
High school diploma or GED	5 (17.3)	8 (25.0)
Some college	3 (10.3)	2 (06.3)
College or graduate degree	7 (24.4)	9 (28.1)
< High school education		

Health Insurance	6 (50.0)	31 (96.9)
Medicaid	1 (08.3)	-
Medicare	5 (41.7)	-
Private	17 (58.6)	1 (03.1)
Uninsured		

Pre-treatment characteristics	17 (59.6)	21 (65.6)
Unemployed	1 (03.4)	26 (81.3)
Homeless or temporary housing		

Ever jailed or incarcerated	27 (92.6)	29 (90.6)

Jail times	5.0 ± 07.2	7.1 ± 09.3

Mandated to treatment	16 (55.1)	19 (59.4)

**Table 2:** Characteristics of residential substance use treatment staff (n = 37)

	Richmond Staff (n = 20)	NY Staff (n = 17)

	*n (%) or M± SD*	*n (%) or M± SD*

Gender	18 (90.0)	8 (47.1)
Female	2 (10.0)	9 (52.9)
Male		

Age (years)	48.4 ± 10.8	51.4 ± 10.6

Race/Ethnicity	14 (70.0)	11 (64.7)
Black/African American	2 (10.0)	1 (05.9)
Hispanic	3 (15.0)	3 (17.6)
White/Caucasian	-	1 (05.9)
Native American	1 (05.0)	1 (05.9)
Other		

Education	1 (05.0)	1 (06.3)
High school diploma/equivalent	3 (15.0)	2 (12.5)
Some college	5 (25.0)	1 (06.3)
Associates degree	6 (30.0)	4 (25.0)
Bachelor’s degree	5 (25.0)	5 (31.3)
Master’s degree	-	3 (18.8)
Doctoral degree		

Discipline	2 (10.0)	-
Nurse/LPN	6 (30.0)	5 (30.0)
Counseling	3 (15.0)	2 (15.0)
Psychologist	3 (15.0)	2 (13.3)
Vocational Rehabilitation	1 (05.0)	2 (05.0)
Social Work	1 (05.0)	1 (05.0)
Administration	1 (05.0)	1 (05.0)
Criminal Justice	1 (05.0)	1 (05.0)
Medical Assistant	-	1 (05.0)
Education	2 (10.0)	2 (10.0)
Other		

Years in Agency	5.3 ± 6.8	7.5 ± 6.0

Years Working in Substance Abuse	10.3 ± 8.1	13.1 ± 6.7

**Table 3:** Thematic analysis of data

	*NYC Clients (N = 32)*		*RVA Clients (N = 29)*		*All Clients (61)*	*NYC Staff (N = 17)*		*RVA Staff (N = 20)*		*All Staff (37)*	*All Urban (N = 49)*	*All Rural (N = 49)*	*All Clients/Staff (N = 98)*
	*n*	%	*n*	%	%	*n*	%	*n*	%	%	%	%	%
Personal Barriers	31	96.9%	20	69.0%	83.6%	16	94.1%	14	70.0%	81.1%	95.9%	69.4%	82.7%
Doubt	10	31.3%	0	0.0%	16.4%	5	29.4%	6	30.0%	29.7%	30.6%*	12.2%*	21.4%
Denial	14	43.8%	9	31.0%	37.7%	4	23.5%	5	25.0%	24.3%	36.7%	28.6%	32.7%
Self-Choice	22	68.8%	11	37.9%	54.1%	9	52.9%	6	30.0%	40.5%	63.3%**	34.7%**	49.0%
Attachment to Alcohol / Drugs	12	37.5%	6	20.7%	29.5%	4	23.5%	5	25.0%	24.3%	32.7%	22.4%	27.6%
Stigma	5	15.6%	1	3.4%	9.8%	5	29.4%	3	15.0%	21.6%	20.4%	8.2%	14.3%
Unaware of Treatment Options	5	15.6%	1	3.4%	9.8%	1	5.9%	4	20.0%	13.5%	12.2%	10.2%	11.2%
Interpersonal Barriers	4	12.5%	3	10.3%	11.5%	3	17.6%	4	20.0%	18.9%	14.3%	14.3%	14.3%
Negative social support	4	12.5%	3	10.3%	11.5%	3	17.6%	4	20.0%	18.9%	14.3%	14.3%	14.3%
Structural Barriers	5	15.6%	7	24.1%	19.7%	3	17.6%	8	40.0%	29.7%	16.3%	30.6%	23.5%
Inaccessibility of Treatment	4	12.5%	4	13.8%	13.1%	1	5.9%	6	30.0%	18.9%	10.2%	20.4%	15.3%
Admission Difficulty	1	3.1%	2	6.9%	4.9%	0	0.0%	3	15.0%	8.1%	2.0%	10.2%	6.1%
Unfeasibility of Treatment	3	9.4%	1	3.4%	6.6%	2	11.8%	3	15.0%	13.5%	10.2%	8.2%	9.2%

* p < 0.05

** p < 0.01.

## Data Availability

The datasets generated and/or analyzed during the current study are available from the corresponding author on reasonable request.

## Abbreviations

SUD: Substance Use Disorder
COD: Co-occurring Substance Use Disorder and Mental Illness

## References

[1] McLellan AT. Have we evaluated addiction treatment correctly? Implications from a chronic care perspective. Addiction. 2002;97(3):249–52. 11964098 10.1046/j.1360-0443.2002.00127.x

[2] National Survey on Drug. Use and health [Internet]. [cited 2023 Nov 13]. Available from: https://www.samhsa.gov/data/data-we-collect/nsduh-national-survey-drug-use-and-health.

[3] Copeland J. A qualitative study of barriers to formal treatment among women who self- managed change in addictive behaviours. J Subst Abuse Treat. 1997;14(2):183–90. 9258863 10.1016/s0740-5472(96)00108-0

[4] Wisdom JP, Cavaleri M, Gogel L, Nacht M. Barriers and facilitators to adolescent drug treatment: Youth, family, and staff reports. Addict Res Theory. 2011;19(2):179–88.

[5] Allen K. Barriers to treatment for addicted African-American women. J Natl Med Assoc. 1995;87(10):751. 7473850 PMC2607901

[6] Priester MA, Browne T, Iachini A, Clone S, DeHart D, Seay KD. Treatment access barriers and disparities among individuals with co-occurring mental health and substance use disorders: an integrative literature review. J Subst Abuse Treat. 2016;61:47–59. 26531892 10.1016/j.jsat.2015.09.006 PMC4695242

[7] Browne T, Priester MA, Clone S, Iachini A, DeHart D, Hock R. Barriers and facilitators to substance use treatment in the rural south: A qualitative study. J Rural Health. 2016;32(1):92–101. 26184098 10.1111/jrh.12129

[8] Barman R, Mahi R, Kumar N, Sharma C, Singh Sidhu K, Singh B, Mittal D. N. Barriers to treatment of substance abuse in a rural population of India. open Addict J. 2011;4(1).

[9] Bobrova N, Rhodes T, Power R, Alcorn R, Neifeld E, Krasiukov N, Latyshevskaia N, Maksimova S. Barriers to accessing drug treatment in Russia: a qualitative study among injecting drug users in two cities. Drug Alcohol Depend. 2006;82:57–63. 10.1016/s0376-8716(06)80010-4 16769447

[10] Kelly BC, Liu T, Zhang G, Hao W, Wang J. Factors related to psychosocial barriers to drug treatment among Chinese drug users. Addict Behav. 2014;39(8):1265–71. 24813554 10.1016/j.addbeh.2014.04.012 PMC4075974

[11] Lieber E, Weisner T, Taylor J. Dedoose software. California: Sociocultural Research Consultants; 2011.

[12] Mcleroy B, Bibeau A, Steckler Glanz. 1988. An ecological perspective on health promotion programs. Health Educ Q;15(4). 10.1177/109019818801500401 3068205

[13] Fisher RA. On the interpretation of χ 2 from contingency tables, and the calculation of P. J Roy Stat Soc. 1922;85(1):87–94.

[14] Ellis B, Bernichon T, Yu P, Roberts T, Herrell JM. Effect of social support on substance. abuse relapse in. a residential treatment setting for women. Eval Program Plan. 2004;27(2):213–21.

